# DR-5 and DLL-4 mAb Functionalized SLNs of Gamma-Secretase Inhibitors- An Approach for TNBC Treatment

**DOI:** 10.34172/apb.2021.070

**Published:** 2020-10-19

**Authors:** Mamta Kumari, Praveen T. Krishnamurthy, Sai kiran S. S. Pinduprolu, Piyongsola Sola

**Affiliations:** Department of Pharmacology, JSS College of Pharmacy, JSS Academy of Higher Education & Research, Ooty, The Nilgiris, Tamil Nadu, India.

**Keywords:** Triple negative breast cancer (TNBC), Death receptor 5 (DR-5), Delta-like ligand 4 (DLL4), Notch Signaling, Gamma secretase inhibitors (GSIs), Dual targeting

## Abstract

Triple-negative breast cancer (TNBC) is the most aggressive and heterogeneous cancer subtypes. High rates of metastasis, poor prognosis, and drug resistance are the major problems associated with TNBC. The current chemotherapeutics eliminate only the bulk tumor cells (non-BCSCs) and do not affect breast cancer stem cells (BCSCs). The BCSCs which are left behind after chemotherapy is reported to promote recurrence and metastasis of TNBC. Death receptor-5 (DR-5) is exclusively expressed in TNBCs and mediates the extrinsic pathway of apoptosis. DR-5, therefore, can be exploited for targeted drug delivery and to induce apoptosis. Gamma-secretase mediated Notch signaling in BCSCs regulates its proliferation, differentiation, and metastasis. The endogenous ligand, Delta-like ligand 4 (DLL4), is reported to activate this Notch signaling in TNBC. Blocking this signaling pathway using both gamma-secretase inhibitors (GSIs) and DLL4 monoclonal antibody (mAb) may produce synergistic benefits. Further, the GSIs (DAPT, LY-411575, RO4929097, MK0752, etc.) suffer from poor bioavailability and off-target side effects such as diarrhea, suppression of lymphopoiesis, headache, hypertension, fatigue, and ventricular dysfunctions. In this hypothesis, we discuss Solid lipid nanoparticles (SLNs) based drug delivery systems containing GSIs and surface modified with DR-5 and DLL4 monoclonal antibodies (mAb) to effectivity target and treat TNBC. The delivery system is designed to deliver the drug cargo precisely to TNBCs through its DR-5 receptors and hence expected to reduce the off-target side effects of GSIs. Further, DLL4 mAb and GSIs are expected to act synergistically to block the Notch signal mediated BCSCs proliferation, differentiation, and metastasis.

## Introduction

Triple-negative breast cancer (TNBC) is characterized by the lack of estrogen receptor (ER), progesterone receptor (PR), and human epidermal growth factor receptor (HER2).^1^ TNBC grows spread, and recur after treatment because of its aggressive pathological features.^2,3^ TNBC accounts for approximately 10–15% of all invasive breast cancer with a short overall survival.^4^ Prevalence of TNBC in India (27% to 35%) is significantly higher compared to western population (12% to 17%).^5^ Existing chemotherapies for TNBC treatment can eradicate the rapidly dividing bulk tumor breast cancer cells (non-BCSCs), and they do not affect small subpopulation of breast cancer stem cells (BCSCs).^6^ Available evidence suggests that the leftover BCSCs are the leading cause of metastasis through epithelial to mesenchymal transition (EMT) process. In the EMT process, the epithelial BCSCs cells lose their inter-cellular adherence by gaining invasion and migration capabilities.^7,8^ There is a need, therefore, to eliminate BCSCs in addition to non-BCSCs.

The Notch signaling pathway is a fundamental regulator of angiogenesis in non-BCSCS and self-renewal & maintenance in BCSCs.^9-11^ The above findings has prompted scientists to evaluate possible benefits of gamma-secretase inhibitors (GSIs) in TNBC to control growth, prevent self-renewal, and suppress drug resistance of TNBCs.^12-16^ Some studies suggest that, although GSIs, like DAPT, LY-411575, RO4929097, MK0752 are not significantly cytotoxic, several studies indicate that they will be useful in potentiating the cytotoxic effects of other anticancer agents and helpful in eliminating BCSCs. However, the GSIs are associated with severe off-target side effects such as diarrhea, suppression of lymphopoiesis, headache, hypertension, fatigue and ventricular dysfunctions, which limit their clinical use.^17-19^ Targeted delivery of GSIs to TNBCs could be one of the strategies to overcome this problem.^15,20^

Activation of Notch1 and 4 receptors by delta-like ligand 4 (DLL4) endogenous ligand in TNBC results in aberrant activation of Notch signaling.^21^ DLL4 binding to Notch1/Notch4 receptors ensures the cleavage of the Notch intracellular domain (NICD) by the enzyme gamma-secretase. The NICD translocates to the nucleus and activates the expression of notch target genes involved in angiogenesis, apoptosis, metastasis, and chemoresistance.^22,23^ DLL4 and gamma-secretase enzyme are highly expressed in TNBC cells and are, therefore, considered as unique targets of TNBC.^24,25^

Recent studies have reported that GSIs are useful in the eradication of BCSCs, inhibition of EMT, angiogenesis, and tumor growth.^19,26,27^ Besides, GSIs also potentiate the effect of chemotherapeutic agents by inhibiting the genes involved in chemoresistance (Hes and Hey).^28,29^ Existing evidence also suggests that the inhibition of DLL4 mediated activation of Notch receptors by anti-DLL4 mAb also produce anti-angiogenic, proapoptotic and chemo-sensitizing effects on TNBC cells.^30-32^ It was recently reported that a combination of DLL4 mAb with GSIs has synergetic proapoptotic effects.^33^

Death receptor 5 (DR-5) is a member of tumor necrosis factor receptor superfamily, overexpressed explicitly on the surface of TNBC cells. Therefore, it can be used as a target for effective drug delivery in TNBCs.^34,35^ Besides, activation of DR-5 leads to activation of extrinsic apoptotic signaling and hence proapoptotic effects.^36^

Solid liquid nanoparticles (SLNs) are widely used for targeted drug delivery to minimize off-target effects and improve the bioavailability of drugs.^37^ Also, SLNs offer several advantages such as improved stability of the drug, higher entrapment efficacy, and biocompatibility over other nanoparticle-based delivery systems. SLNs with appropriate stealth properties will have reduced clearance and improved drug cargo delivery to the tumor site through enhanced permeation and retention (EPR) effect.^38,39^ However, accumulating evidence suggest that the EPR effect alone` is not sufficient to achieve site-specific delivery of drug cargo to the tumor site. Alternatively, unique cancer surface proteins have been targeted using monoclonal antibodies (mAbs) to improve target-specific delivery of drug cargo.^40^

## Hypothesis

TNBC targeted GSI-SLNs surface modified with DR-5, and DLL4 mAbs can be an effective strategy to treat TNBC.

The SLN delivery system will deliver the GSIs drug cargo precisely to TNBCs through membrane DR-5 receptors and hence reduce the off-target side effects of GSIs. DLL4 mAb and GSIs are expected to act synergistically to block the Notch signal mediated BCSCs proliferation, differentiation, and metastasis. Further, when combined conventional chemotherapy, the formulation will effectively eliminate both BCSCs and non-BCSCs and, thus, help in achieving the complete cure of TNBC.

## Explanation of the hypothesis

**Figure 1:**
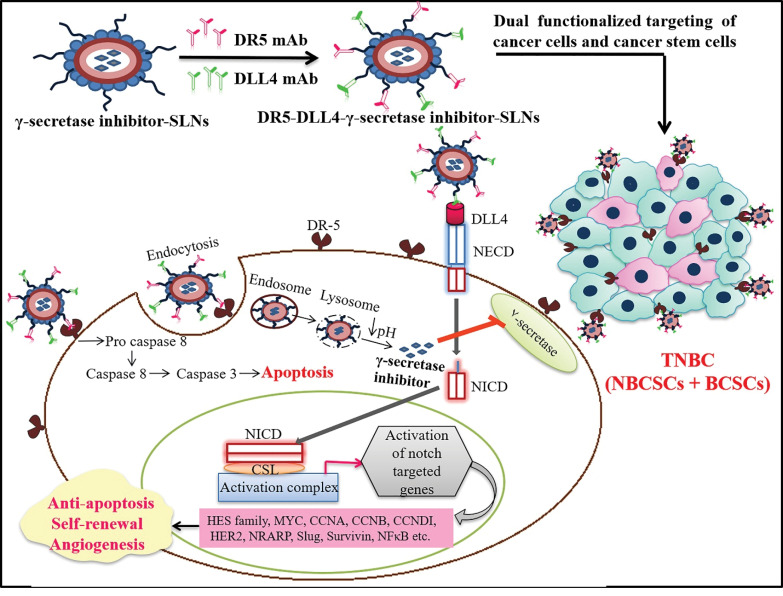
SLNs of Gamma-secretase inhibitor (GSI) surface modified with dual mAbs for the site specific delivery of GSI to breast cancer cells and breast Cancer Stem Cells (BCSCs). Silencing of Notch signaling by this apporach inhibits cell proliferation, angiogenesis, apoptotic resistance and self-renewal of BCSCs.

Notch signaling is one of the critical pathological pathways implicated in TNBCs. This pathway regulates the expression of target genes such as HES1, HEY2, MYC, CCND1, HES4, NRAR, etc., and involved in BCSCs proliferation, differentiation, and apoptosis.^41,42^ Notch signaling is activated by the binding of transmembrane ligands [Delta-like (DLL) 1, 4, and Jagged (JAG) 1, 2] to the Notch receptors present on the cell surface. This binding results in the proteolytic invasion of Notch by a presenilin-dependent gamma-secretase complex to release of NICD, which later translocates to the nucleus and heterodimerizes with a transcription factor, CSL (suppressor of hairless), and activates various target genes (Figure 1).^43-45^ Several studies conclude that Notch signaling pathway activation in BCSCs predominantly activates the expression of HES1 and HEY2 genes, which participates in the perpetuation of self-renewal of BCSCs.^46-48^ Notch signaling also reported to regulate cyclinD1, c-Myc, p21, Survivin, Slug, CCNA, CCNB, CCNDI, HER2 expression, and stimulate the nuclear factor-kappa B (NF-κB) pathway.^49,50^ The Notch-mediated activation of all these factors promotes cell proliferation, angiogenesis, resistance to apoptosis, and self-renewal of BCSCs.

Surface modified SLNs with DR-5 mAb expected to bind selectively to DR-5 receptors present on TNBC and therefore help in active targeting through receptor-mediated endocytosis, further binding with DR-5 may also initiate the extrinsic pathway of apoptosis and hence proapoptotic effects.^51^ Ding et al demonstrated that anti-DR-5 mAb-mediated delivery of dacarbazine (DTIC) nanoparticles show an improved antitumor and proapoptotic activity when compared to the bulk drug.^52^ Tummala et al, report that, gold nanoparticles of oxaliplatin conjugating with anti-DR-5 mAb show enhanced anticancer activity and site-specific delivery of drug cargo.^53^ Zhang et al reported that anti-DR-5 (Zaptuzumab) antibody-drug conjugate show increased therapeutic efficacy and safety when compared to naked antibody Zaptuzumab treatment.^54^

The expression of Notch ligand (DLL4) in tumor vasculature is high as compared to healthy tissues.^55^ In TNBC, DLL4 binding to the Notch receptors promotes the transcription activation of genes regulating tumor angiogenesis and growth.^55,56^ DLL4 mediated activation of Notch signaling, therefore, improves vascular function and promotes tumor growth.^57^ Jia et al demonstrated that a humanized anti-DLL4 mAb inhibits breast tumor growth in an MDA-MB-231 xenograft model in mice. The results indicate that anti-DLL4 mAb prevents tumor growth by blocking the DLL4-Notch signaling pathway.^58^ Hoey et al report that anti-DLL4 treatment blocks the Notch pathway mediated expression of anti-apoptotic genes (HSPA6 and BIRC3) and hence sensitize the tumor cells towards chemotherapy.^59^

Yen et al establish that the combination of anti-DLL4 antibody and paclitaxel decrease BCSCs frequency and retard the tumor relapse in paclitaxel-resistant TNBCs.^60^ Wang et al successfully developed antibody-drug conjugates of Monomethyl auristatin E and anti-DLL4 antibody to promote tumor cell death and to achieve site-specific delivery.^61^

Gamma-secretase enzyme regulates tumor-promoting Notch signaling in TNBC by controlling NICD levels. Inhibition of this enzyme through GSIs, therefore, a promising strategy.^62,63^ Accumulating pieces of evidence suggest that, the GSIs are associated with clinical limitations such as poor bioavailability and off-target side effects include diarrhea, suppression of lymphopoiesis, headache, hypertension, fatigue, and ventricular dysfunctions.^17,19,63^ To overcome these limitations, researchers focused on nanocarriers based drug delivery which improves bioavailability and delivers the drug cargo specifically to tumor sites.^15,64^

Mamaeva et al developed a glucose functionalized Mesoporous silica nanoparticles of GSI (DAPT). They report that the developed nanoformulation efficiently delivered the drug to TNBC and reduced the BCSCs population by inhibiting the Notch signaling pathway.^15^ Kang et al establish that the concurrent treatment of GSIs with anti-DLL4 increases the anticancer and proapoptotic efficiency of GSIs in gastrointestinal cancer. The combined therapy of GSIs with anti-DLL4 boosted the expression of BAX and P53 and reduced the expression of Bcl-2. On the other hand, naïve GSI only enhanced the expression of BAX and P53, suggesting that the reduced Bcl-2 expression had a significant function in synergistic antitumor and proapoptotic effects.^33^

Drug delivery using SLNs is an accepted approach for targeted drug delivery to improve efficacy and reduce off-target side effects. SLNs as a nanocarrier, enhance the bioavailability, and provide control release. Besides, SLNs also modulate the release kinetics, minimize systemic toxicity and increase the therapeutic efficacy of chemotherapeutic agents.^65,66^ Pindiprolu et al reported that, SLNs of niclosamide improve the anticancer efficiency by increasing the site-specific delivery of niclosamide to the TNBC.^67^ Wang et al successfully demonstrate the improved anticancer efficacy of Curcumin SLNs in SKBR3 cells as compared to naïve curcumin.^68^

Dominguez et al successfully conjugated anti-RNEU and anti-CD40 antibodies on the surface of PLA-(poly dl-lactic acid)-biodegradable nanoparticles. They establish that dual antibody conjugated nanoformulation boosts the antitumor response and also results in complete tumor eradication.^69^ Kosmides et al developed nanoparticles surface modified with two diﬀerent mAbs (anti-PD-1 and anti-CTLA-4 mAb), which concurrently impede the inhibitory PD-L1 signal and activate T cells through the 4-1BB co-stimulatory pathway.^70^ The combination of mAbs on the surface of nanoparticles has significantly increased their efficacy when compared to individual mAbs.^70^ Chen et al developed dual antibodies (anti-CD44 and anti-CD133) conjugated transretinoic acid-loaded poly(lactide-co-glycolide)-lecithin-PEG nanoparticles to target cancer stem cells (CSCs). They report that dual-targeted nanoparticles effectively inhibit the tumor growth and eradicate the CSC population.^71^

## Conclusion

In this article, we propose to prepare DR-5, and DLL-4 mAbs functionalized SLNs of GSIs to enhance their bioavailability, provide TNBC specific delivery, and to reduce off-target side effects. Further, anti-DLL-4 mAb and GSIs may synergistically act to eliminate the resistant BCSCs of TNBC. Besides, when combined with anticancer chemotherapeutics, the formulation may enhance their overall anticancer efficacy.

## Ethical Issues

The study does not involve any human subjects and/or animals.

## Conflict of Interest

The authors confirm that this article content has no conflict of interest.

## Acknowledgments

The authors would like to thank the Department of Science and Technology- Fund for Improvement of Science and Technology Infrastructure in Universities and Higher Educational Institutions (DST-FIST), New Delhi, for their infrastructure support to our department (Grant No. SR/FST/LSI-574/2013). MK acknowledges AICTE-NDF (Application No.- 52198) for her SRF.
